# Frequent callers vs. frequent users – a scoping review of frequent contacts to the emergency medical services

**DOI:** 10.1186/s12245-025-00925-0

**Published:** 2025-06-20

**Authors:** Astrid Karina Valås Harring, Maria Kjærgaard, Tine Bennedsen Gehrt

**Affiliations:** 1 Institute for Nursing and Health Promotion, OsloMet, Oslo Norway; 2 Division of Prehospital Services, Oslo University Hospital, Oslo, Norway; 3 Department of Research and Development, Prehospital Emergency Medical Services, Central Denmark Region, Aarhus, Denmark; 4 Department of Clinical Medicine, Aarhus University, Aarhus, Denmark

**Keywords:** Emergency medical communication centre, Emergency medical services, Frequent caller, Prehospital

## Abstract

**Background:**

A significant limitation in the literature on frequent callers and frequent users of prehospital Emergency Medical Services (EMS) is the lack of consistent and thus, comparable definitions, as well as inconsistent use of terminology. Here we aim to summarise and address discrepancies in the existing literature, contributing to the ongoing discussion.

**Method:**

We conducted a systematic search of available literature from 2000 up until February 2024 in the PubMed database. Search terms related to both frequent *callers* and frequent *users* of the prehospital EMS.

**Result:**

A total of 19 articles were included in our analysis of definitions of frequent callers and users of prehospital EMS. The average minimum number of calls required to be defined as a frequent *caller* was 42.5 calls per year (range: 4-120). For frequent *users*, an average minimum number of ambulance responses was 4.7 per year (range: 3–10).

**Conclusion:**

Our findings emphasise both the possibility and the need to distinguish between frequent *users* and frequent *callers* of prehospital EMS. Existing definitions in the literature vary widely, making comparisons difficult. Standardised definitions and consistent terminology are essential to address underlying issues and enable further research synthesis.

**Supplementary Information:**

The online version contains supplementary material available at 10.1186/s12245-025-00925-0.

## Introduction

The prehospital Emergency Medical Services (EMS) main responsibility is responding to citizens experiencing acute, severe, or potentially life-threatening illness or trauma. Yet, some citizens are frequently in contact with the prehospital EMS; either through emergency calls or through being attended to by an ambulance or other prehospital unit. In the literature, both frequent *users* of and frequent *callers* to the prehospital EMS are described [1], but often interchangeably and without a clear distinction between the terms. However, we find this distinction to be critical, as it forms the basis for the study population and, thus, how we understand and address underlying issues.

Frequent *callers* represent an unsorted group of citizens defined by their more frequent calls to the prehospital EMS compared to other citizens. The term “chronic caller” emerged in the medical literature in the 1970 s [2], and has since evolved into “frequent callers”, a term used in many contexts within healthcare, such as telephone helplines, primary healthcare, specialist medicine clinics, and EMS [3]. Calls to the medical emergency number are accessible to all citizens, at all times, and there are no restrictions on how frequently one may contact the service. Hence, frequent callers is a group of citizens that is not selected based on who meets the system’s criteria or is considered eligible for prehospital assistance, nor are they selected based on the subsequent course of action (e.g., dispatch of prehospital resource). Instead, they are selected by their frequent self-assessed need to make contact. In addition to citizens calling on their own behalf, Harring et al. [4] found that the number of emergency contacts doubled when including calls made by others, such as next of kin, healthcare personnel, and members of the public, who frequently contacted emergency services on behalf of these citizens.

Frequent *users* of the prehospital EMS refer to citizens who frequently have an ambulance or other prehospital unit dispatched or who recurrently are transported by ambulance. That is, this is a selected group that has been assessed to be eligible for prehospital assistance, and contacts (calls) that do not lead to the deployment of EMS resources are excluded and are not considered in this definition. Frequent ambulance use has been linked to chronic health conditions and a high incidence of comorbidities [5].

The prehospital EMS is a distinct component of the emergency healthcare system, which differs in both function and user groups from other parts such as emergency departments. For example, prehospital services provide direct access to the healthcare system for citizens, whereas emergency departments, in some countries and contexts, require referral [6]. Studies linking EMS and hospital data have been undertaken [7–9], and show great promise, but the overlap between the populations is yet to be determined. Hence, in the present scoping review, the focus is exclusively on frequent callers and frequent users of prehospital EMS and not other parts of the emergency healthcare system.

The present scoping review aims to summarise and raise awareness of the discrepancies in the existing literature on frequent *callers* and frequent *users* of prehospital EMS, paving the way for a more conscious use of terminology and definitions.

## Method

In the present study, frequent *callers* were defined as citizens who frequently call the (medical) emergency number, regardless of whether prehospital resources are dispatched or not. Frequent *users* were defined as citizens who frequently have an ambulance or other prehospital EMS resource dispatched, excluding contacts that do *not* result in the deployment of EMS resources, thus always representing a subset of contacts that result in EMS deployment. Hence, frequent users will always be represented within the group of frequent callers, whereas the reverse is not the case.

### Search strategy

**Fig. 1:**
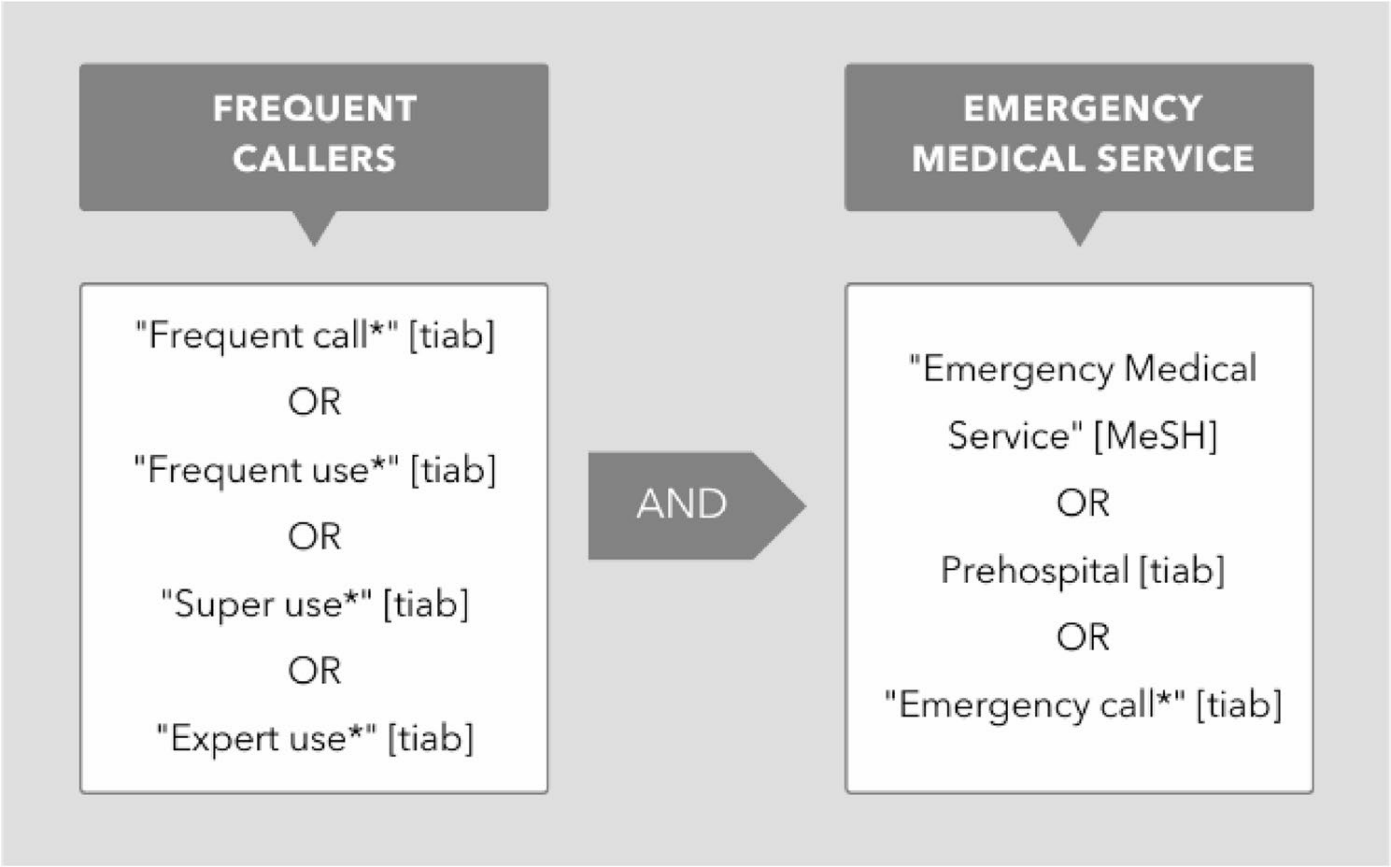
Systematic literature search

The study was not pre-registered but adheres to the JBI (Joanna Briggs Institute) protocol template for scoping reviews. We conducted a systematic search of available literature from January 2000 up until February 2024 in the PubMed database. Search terms related to both frequent *callers* and frequent *users* of the prehospital EMS. The terms were separated in two blocks by the Boolean operators OR within each block and AND between the blocks (Fig. 1, Supplementary material 1). The MeSH tool was used to search within topic hierarchies and for one of the search terms in the final search string, due to an extended hierarchy of subject terms. Truncation (*) was applied to account for variations in the form of the word, and the title/abstract tool was used to ensure that the search terms appeared in these sections. The search was limited to articles written in Danish or English.

Simultaneously with the reading of the selected articles, a citation search was conducted to identify articles not previously detected. This method was applied both retrospectively, by reviewing the reference lists of included articles, and prospectively, by screening articles that cited the included articles.

### Inclusion

**Fig. 2:**
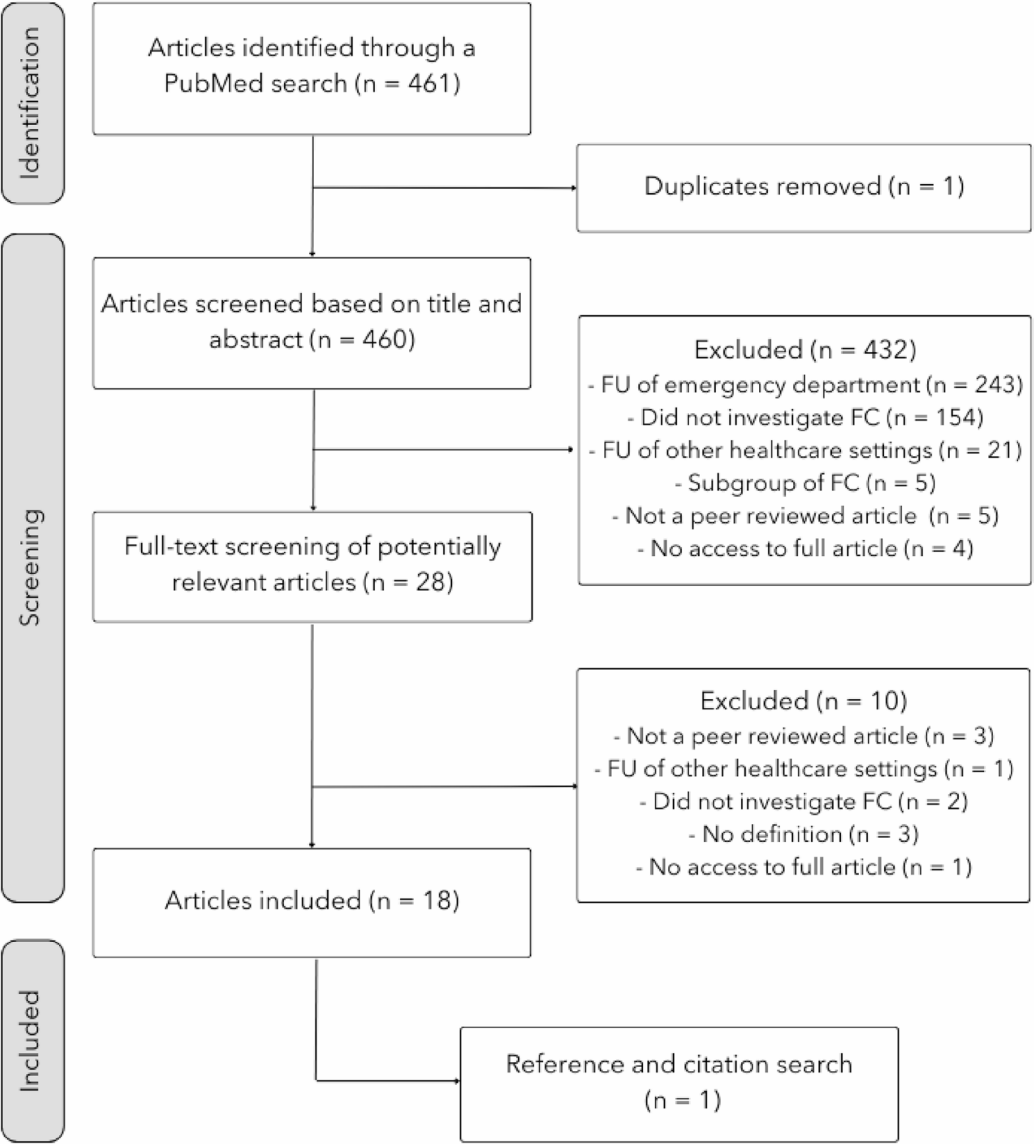
Flow chart of literature search FU = Frequent users, FC = Frequent callers

Figure 2 illustrates the search process. The articles were reviewed by the second author based on their title and abstract to assess whether they met the inclusion criteria. An article was considered eligible for inclusion if it (1) focused on frequent *callers* or frequent *users* of prehospital services, identified either through calls to the medical emergency number or through the use of ambulance services, and (2) provided a frequency or definition of what is required to be considered a frequent caller or frequent user of the prehospital EMS. Hence, the inclusion criteria were broad in an effort to capture any quantifiable definition of frequent *callers* or frequent *users* of prehospital services in the existing literature. Only peer-reviewed articles were included in the final analysis.

### Exclusion

All articles that primarily addressed emergency department use were excluded. Articles that focused exclusively on specific subpopulations within the prehospital EMS were excluded (e.g., elderly patients, patients with specific symptoms or diagnoses), as the interest in the present search was in the unsorted and broad group of frequent *callers* to and *users* of prehospital EMS.

### Source of evidence selection

In total, 461 articles were identified, including one duplicate, that was removed prior to screening (see Fig. 2). In addition, the chain search yielded one previously undiscovered article that matched the inclusion and exclusion criteria.

### Data extraction

Following the PRISMA Extension for Scoping Reviews [10] (see Supplementary material 2), the articles were screened for their chosen quantitative definition i.e. frequency by the second author in close collaboration with the last author. Data was extracted using a standardised extraction template developed by the research team (template: authors, publication year, frequent user or frequent caller, minimum frequency per year). To make comparisons possible, frequency per year was chosen. If not stated explicitly, the yearly frequency was calculated.

### Quality assessment

As our aim was limited to the frequency threshold for being defined as a frequent caller or frequent user, the quality of the included articles was not evaluated. Although formal risk of bias assessment was not performed, in accordance with scoping review methodology, we limited the inclusion to peer-reviewed articles and ensured consistent, systematic extraction across all sources to minimise subjective bias in the interpretation of findings.

## Results

Out of the 460 articles reviewed, 28 were selected for full-text screening, of which 18 met the inclusion and exclusion criteria. The significant reduction was due, among other factors, to the term ‘emergency department’ being included (see Fig. 2). Including the additional article found through citation/chain search, a total of 19 articles were included in our analysis of definitions of frequent callers and frequent users of prehospital EMS.

The 19 included articles were systematically reviewed to identify the definitions of frequent callers and frequent users. The definitions were sorted into two categories in Table 1: calls (defining frequent *callers*) and ambulance responses (defining frequent *users*).

**Table 1:** Definitions of frequent callers and frequent users of prehospital services in published articles

Authors	Year	Country	Definition	Calls per year⁺	Ambulance responses per year⁺
Weiss et al. [11]	2005	USA	≥ 3 calls per month	36	-
Rinke et al. [12]	2012	USA	The 25 citizens with the highest call frequency	n/a	-
Tadros et al. [13]	2012	USA	≥ 10 ambulance responses per year	-	10
Knowlton et al. [14]	2013	USA	≥ 6 ambulance responses in ∼2 years	-	3
Scott et al. [15]	2014	UK	The 100 citizens with the highest call frequency	78^*^	-
Edwards et al. [16]	2015	UK	≥ 10 calls per month	120	-
Hall et al. [17]	2015	USA	≥ 5 ambulance responses per year	-	5
Norman et al. [18]	2016	USA	≥ 4 ambulance responses per year	-	4
Tangherlini et al. [19]	2016	USA	≥ 4 ambulance responses per month over several months	48	-
Tärnqvist et al. [20]	2017	Sweden	≥ 4 ambulance responses per year	-	4
Agarwal et al. [21]	2019	Canada	≥ 5 calls per year	5	-
Kuek et al. [22]	2019	Singapore	≥ 4 calls per year	4	-
Snooks et al. [23]	2019	UK	≥ 5 calls per month or; ≥ 12 calls in three months	60 or; 48	-
Søvsø et al. [5]	2019	Denmark	> 2 ambulance responses per year		3
Mahmuda et al. [24]	2020	Canada	≥ 5 calls per year	5	-
Maruster et al. [8]	2020	The Netherlands	≥ 4 calls per year	4	-
Maruster et al. [9]	2021	The Netherlands	≥ 4 ambulance responses per year	-	4
Boland et al. [25]	2023	USA	≥ 3 calls in three months	12	-
Scott et al. [26]	2023	UK	≥ 5 calls per month and ≥ 6 calls per month	60 or; 72	-

⁺Calculated minimum number of calls/ambulance responses on an annual basis based on the concerned definition

^*^Average calls per year for the concerned citizens

For frequent *callers*, an average minimum number of annual calls was calculated from the 12 included definitions. Based on the articles reviewed, the average minimum number of calls required to be defined as a frequent caller was 42.5 calls per year, with a range from a minimum of 4 to 120 calls per year (see Table 1).

For frequent *users*, an average minimum number of ambulance responses per year was calculated based on definitions from the seven included studies. This average came to 4.7, ranging from 3 to 10 ambulance responses per year (see Table 1).

## Discussion

### Summary of key findings

The present scoping review examined thresholds for being considered a frequent *caller* and frequent *user* of prehospital EMS in the existing literature. The majority of the identified studies focused on frequent use of emergency departments, where there is a higher degree of consistency in the definitions of frequent contacts, enabling comparison across studies [1]. In contrast, research in the prehospital context is more widespread, and we found a significant discrepancy in the available literature regarding definitions of both frequent *users* and frequent *callers*, and less consistent use of terminology distinguishing the two types of contact. Our findings highlight the importance of distinguishing between frequent *users* and frequent *callers* to the prehospital EMS and for standardising definitions of when contacts are considered ‘frequent’.

#### Vast differences in thresholds

The results demonstrated vastly different thresholds for the number of prehospital contacts per year to be considered a frequent caller *versus* a frequent user and for being considered a frequent caller or frequent user *across studies*.

Definitions of frequent *callers* varied from a minimum of four to a maximum of 120 calls per year. This inconsistency suggests a pragmatic approach to definitions, which often lack well-founded arguments. In some cases, definitions are not based on existing literature, and in others, they fail to reflect empirical data. There was more consistency in the definition of frequent *users* across studies, suggesting more agreement across studies of what constitutes *frequent* EMS use.

The findings underscore that frequent callers and frequent users are defined and treated differently across the literature, with implications for comparability and intervention design. The broader variation in call-based definitions suggests less consensus and more heterogeneity in this group, likely due to differences in who initiates contact and how calls are triaged. In contrast, frequent users are consistently defined based on eligibility criteria defined by the EMS system, potentially leading to narrower thresholds.

#### Timeframes

Another issue in the existing research is the short timeframes used in many studies. Several studies use data covering only a few months or set criteria based on calls e.g. per month, which can be problematic as it identifies frequent contacts based on a snapshot in time. Individuals experiencing short-term illness or a temporary exacerbation of a chronic condition, resulting in an unusually high call or use frequency during that period, may not be part of the group that places sustained pressure on the system. By using contact data from a longer time period (e.g., an entire year), it is possible to identify citizens who make consistently frequent use of prehospital services.

The large variation in both the criteria for the number of contacts (particularly calls) and the timeframes used makes it difficult to identify patterns across studies and creates poor conditions for conducting meta-analyses. This is a major limitation in existing research, as studies are less generalisable and harder to compare on a broader scale.

#### Relative frequency

All definitions of frequent *callers* and frequent *users* of the prehospital EMS identified in the present scoping review used a specific number of annual calls for defining these groups of citizens, except two studies that examined a specific sample of frequent callers or users (e.g., the 100 most frequent callers [15]).

It is important to note that this scoping review aimed to determine the average minimum number of calls required to be defined as a frequent caller, thus the number represents only the entry threshold for inclusion in each study and does not reflect actual mean or median call volumes within study populations. The true average number of calls made by individuals classified as frequent callers may therefore be higher. The same regards frequent users. Table 1 reports the minimum criteria used for categorisation and do not capture the distribution or mean number of responses among those categorised as frequent users or frequent callers.

Using a specific number of annual calls for the definition of frequent *callers* might not be applicable to all services and across countries due to differences in how emergency call systems are structured and how calls are recorded. For example, in Norway citizens call directly to the *medical* emergency number, whereas for most of Europe (including Denmark) and the USA there is a joint emergency number (e.g., covering police, fire brigade, and prehospital EMS). This is an important difference when ‘counting calls’ as for some prehospital services, calls are screened and filtered, and hence, categorised as ‘medical’ or ‘non-medical’ by staff at the joint emergency number before calls reach the prehospital EMS. For example, the most frequent caller in Oslo, Norway, had more than 3000 calls in one year, while in the Central Denmark Region, Denmark, where there is pre-selection by the police, the highest call frequency was 150 calls in 2022. Such organisational differences might be why some previous studies used 4 calls per year and others used 120 annual calls as the threshold for being defined as a frequent *caller* (see Table 1). However, both may be a high frequency relative to all calls made to the EMS from which the data originated. These structural and procedural differences introduce variability in what counts as a ‘call’, how calls are logged in EMS data systems, and ultimately, how frequent calling is defined or detected in research across countries.

For frequent *users*, applying a specific number of annual dispatches of prehospital units (e.g. ambulances) for defining this group may be more applicable across services. This could be one explanation for the greater consistency in definitions of frequent users across studies which only ranged from 3 to 10 EMS responses per year. This consistency could also reflect that frequent *users* of prehospital EMS (compared to the unselected group of frequent *callers*), are selected by ‘the healthcare system’ as being eligible for prehospital assistance, and this selection may show little variation across services and countries, resulting in similar conceptions of what constitutes *frequent* EMS use. Many frequent *users* are already in contact with healthcare personnel, who facilitate their frequent ambulance transfers to, from, or between healthcare services [4]. Hence, frequent users of prehospital resources might differ from frequent callers in important ways, for example by already being in contact with healthcare personnel who facilitate the frequent contacts to the prehospital EMS for them. This may suggest a structural distinction, whereby frequent users may more often be part of formal pathways of care, while frequent callers may rely more on self-initiated contact. However, we do not suggest that frequent callers necessarily lack healthcare needs. Some frequent callers may have similar health burdens as frequent users but face barriers to e.g. navigating the healthcare system. These potential disparities warrant further studies.

#### Inconsistent use of terminology

Another major limitation of the existing literature is the inconsistent use of terminology distinguishing between the two types of contact (calls and use) with the prehospital EMS, thus ‘frequent contacts’ have recently been proposed to encompass both [4]. We urge researchers and auditors to think of frequent contacts to the prehospital EMS as a spectrum, where a citizen can be placed, and to consider their contacts as something that can generate an EMS response - rarely, sometimes or often.

### Context of existing research evidence

This is the first review of the literature concerning definitions of frequent *callers* to and *users* of the prehospital EMS. Furthermore, it is the first to address both the vast variety within and between the two types of contacts.

Our search results are consistent with prior reviews on this topic. A systematic review by Scott et al. [1] on frequent callers and users from 1983 to 2012 included 44 articles, followed by Skogevall et al. [3] who conducted an integrative review on frequent callers, between 2011 and 2020, and included 20 studies. As seen by our Fig. 2, we included 19, as some were excluded based on lack of peer review and not providing a definition.

The various frequencies within the definitions represent a significant limitation in the literature and our findings contribute to standardising future research on this topic in the prehospital setting, in order to a gain better understanding and develop systems to identify these individuals. Future studies should examine the applicability of definitions in other prehospital contexts and countries, where patterns and causes of frequent contact may differ. Such studies should also include call data for longer periods of time (e.g. over an entire year) to maintain a long-term perspective. Also, it is relevant to further examine sub-groups of frequent callers and frequent users and the effect of factors such as socioeconomic status, chronic illness, comorbidity, municipal support, psychiatric diagnoses, etc. on the frequent contacts.

### Strengths and limitations

Studies primarily focusing on the emergency department were excluded in the title and abstract screening. Although this decision aligns with the intention to focus specifically on prehospital EMS populations, we recognise that this exclusion may overlook important overlaps, particularly among frequent EMS users who commonly transition between EMS and emergency department settings. The initial review was performed by only the second author and was limited to one database (PubMed). However, the following citation chain search mitigates this limitation. Limiting the final inclusion to peer-reviewed articles excludes e.g. commentaries, opinion pieces, internal reports. As disclosed in the methods, we did not evaluate the quality of the included studies, as we only extracted the minimum number of contacts to be considered a frequent caller or frequent user from the study. Hence, study or data quality was not a concern for including studies and no quality assessment was performed.

### Implications for future research, policy and practice

In order to relieve the pressure put on EMS systems by frequent contacts, we consider the distinction between frequent *callers* and frequent *users* essential, as the underlying reasons and subsequent needs may be very different. Further studies should examine demographic, clinical, and socioeconomic characteristics, such as chronic disease burden, mental health, housing instability, or lack of social support, that may underlie frequent contacts to the EMS. Such information may be used to identify appropriate measures for the citizen and, with further research, support the prediction of suitable future interventions based on their circumstances, highlighting the need for a systematic approach and guidelines that enable multidisciplinary cooperation and support an individualized approach [3]. A review of potentially effective strategies has recently been conducted by Jones et al. [27]. However, identification and eligibility for interventions depends on whether individuals meet an established criteria, underscoring the importance of work that goes into formulating a definition and setting a frequency for *frequent* contacts.

There is a pressing need to agree on operational definitions for *frequent caller*s and *frequent users* of the EMS that are applicable across services and countries. A formal consensus-building process, such as a Delphi study, could be an appropriate next step. This would improve comparability between studies and support the development of evidence-based interventions.

## Conclusion

There are various proposed definitions of frequent *callers* to and frequent *users* of the prehospital EMS in the literature, however these definitions are often not comparable, and there is considerable variation in what is considered ‘frequent’ and what terminology is applied. There is a pertinent need for standardised definitions to classify frequent *callers* and frequent *users* and to streamline the terminology. This is crucial for understanding and addressing the underlying issues, and for paving the way for research synthesis and effective interventions. It is important, however, to recognise the complexity of the reasons for frequent contact to the prehospital EMS, which may differ between regions, countries, and healthcare systems.

## Supplementary Information

Supplementary Material 1.

Supplementary Material 2.

## Data Availability

No datasets were generated or analysed during the current study.

## Abbreviations

EMCC: Emergency medical communication centre
EMS: Emergency medical services
