# Supplementary material for: Frequent callers vs. frequent users – a scoping review of frequent contacts to the emergency medical services
Source: Int J Emerg Med. 2025 Jun 20;18:108. doi: 10.1186/s12245-025-00925-0 (PMC12180229; doi:10.1186/s12245-025-00925-0)
Supplement: Supplementary file 1 — Supplementary Material 1. [file 12245_2025_925_MOESM1_ESM.docx]

Supplementary material 1: Search strategy

|  | PubMed |
| --- | --- |
| 1 | “Frequent call*" OR "Frequent use*" OR "Super use*" OR "Expert use*" |
| 2 | "Emergency Medical Service" OR “Prehospital” OR "Emergency call*" |
| Frequent users and callers | 1 AND 2 |
